# Shift in energy metabolism caused by glucocorticoids enhances the effect of cytotoxic anti-cancer drugs against acute lymphoblastic leukemia cells

**DOI:** 10.18632/oncotarget.21689

**Published:** 2017-10-09

**Authors:** Shigeki Aoki, Michie Morita, Takuya Hirao, Masashi Yamaguchi, Reika Shiratori, Megumi Kikuya, Hiroji Chibana, Kousei Ito

**Affiliations:** ^1^ Laboratory of Biopharmaceutics, Graduate School of Pharmaceutical Sciences, Chiba University, Chiba-city, Chiba 260-8675, Japan; ^2^ Medical Mycology Research Center, Chiba University, Chiba-city, Chiba 260-8673, Japan

**Keywords:** ALL, autophagy, glucocorticoids, glycolysis, oxidative phosphorylation

## Abstract

Acute lymphoblastic leukemia (ALL) is the most common childhood malignancy. Treatments include glucocorticoids (GCs) such as dexamethasone (Dex) and prednisolone, which may be of value when used alongside cytotoxic anti-cancer drugs. To predict therapeutic efficacy of GCs, their activity against ALL cells is usually examined prior to chemotherapy; however, few studies have examined their effects when used in combination with other drugs. The paradox is that cytotoxic anti-cancer drugs that are effective against proliferating cancer cells show synergistic effects when used with GCs that prevent cell proliferation. To address this point, we investigated intracellular energy metabolism in ALL CCRF-CEM cell clones classified according to their sensitivity to Dex and cytotoxic anti-cancer drugs in bulk cultures of mixed cells. We found that Dex suppressed glycolysis, the most important metabolic system in cancer cells, in cells that were damaged by etoposide (a cytotoxic anti-cancer drug), and the cells showed a concomitant increase in mitochondrial oxidative phosphorylation. Furthermore, autophagy, an intracellular bulk degradation system, regulated mitochondrial viability. We also found that mitochondria, whose function is enhanced by Dex, were susceptible to anti-cancer drugs that inhibit respiratory complexes (e.g., etoposide and daunorubicin), resulting in increased production of reactive oxygen species and subsequent cytotoxicity. Taken together, the present study points the way toward a more accurate prediction of the sensitivity of ALL cells to the combined action of anti-cancer drugs and GCs, by taking into consideration the shift in intracellular energy metabolism caused by GCs: namely, from glycolysis to mitochondrial oxidative phosphorylation mediated by autophagy.

## INTRODUCTION

Acute lymphoblastic leukemia (ALL), one of the most prevalent childhood malignancies, is caused by transformation of immature lymphoid cell-like precursor T or B lymphocytes [1]. While polymorphic variations in several genes, including IKZF1 (at 7p12. 2), ARIDB5 (at 10q21. 2), and CEBPE (at 14q11. 2), increase the risk of developing B cell childhood ALL [2], the underlying mechanism remains unclear. However, the availability of effective anti-cancer drugs and other anti-cancer treatments over the past few decades mean that the overall survival rate is nearly 80% [3]. In particular, glucocorticoids (GCs) (dexamethasone (Dex) and prednisolone) are of great value, particularly when used in combination with cytotoxic anti-cancer reagents such as vincristine, vinblastine, doxorubicin, daunorubicin, and etoposide [4]. GCs exert suppressive effects on lymphocytes by inhibiting cell proliferation via induction of cell cycle arrest at G_1_-phase rather than through cytotoxic mechanisms [5]. Notably however, the effects vary between individuals [6]. At the beginning of ALL treatment, patients are divided into two groups based on their response to GCs: good-responders and poor-responders [7]. Ninety percent of patients fall into the good responder group and have a high cure rate (>80%). Patients in the poor (or inadequate) responder group have an unfavorable outcome, with a probable event-free survival of <50% [7, 8]. Therefore, individual differences in terms of responses to GCs are important determinants of the efficacy of cancer chemotherapy in ALL patients.

To date, reduced reactivity to GCs (associated with genetic mutations or unique isoforms of GCs receptors) [9, 10] and blunted GC-mediated cytostatic effects (linked to an abnormal increase in the rate of glycolysis) [11] have been identified as mechanisms underlying resistance to GCs. However, some patients have an unsatisfactory clinical outcome even after combination chemotherapy with GCs and cytotoxic drugs, despite showing a clinically good response to GCs. Indeed, Kaspers et al. stated that no conclusions regarding prognostic impact could be drawn based on GC responses alone [12]. Furthermore, the authors suggested that, in addition to GC therapy, the response of children with ALL to anti-cancer agents (asparaginase and vincristine) is related significantly to long-term clinical outcome [13]. However, many therapeutic regimens and combinations of anti-cancer drugs are used to treat ALL; therefore, it is impractical to conduct response tests for each individual patient. However, a paradox exists: although GCs have cytostatic effects, most anti-cancer drugs used in combination therapy with GCs are cytotoxic (i.e., they inhibit proliferation or induce apoptosis of abnormally proliferating cancer cells) [14]. Thus, we wondered whether combining GCs with cytotoxic anti-cancer drugs might actually weaken the effects of the latter.

Previous studies show that GCs inhibit the glycolytic pathway in ALL cells by restricting glucose uptake via glucose transporters [15] and by suppressing expression of pyruvate kinase, a key glycolytic enzyme [16]. ALL cells, similar to cancer cells in general, show increased glucose uptake and glucose dependence, which requires a rich supply of ATP via the glycolytic pathway [17]. Thus, cancer-specific energy metabolism pathways might be a target for cancer chemotherapy; for example, 2-deoxyglucose (a hexokinase inhibitor) and 3-bromopyruvate (a dual inhibitor of hexokinase and glyceraldehyde 3-phosphate dehydrogenase) suppress proliferation of cancer cells *in vitro* by inhibiting the glycolytic pathway [18, 19]. However, we should point out that inhibitors of glycolytic enzymes do not show strong anti-cancer effects when used as a single agent *in vivo* [20]. By contrast, glycolytic inhibition by 2-deoxyglucose increases the efficacy of cytotoxic anti-cancer drugs (adriamycin and paclitaxel) in patients with osteosarcoma and non-small cell lung cancer [20]. This may explain why GCs enhance the therapeutic effects of cytotoxic drugs when used in combination chemotherapy regimens. Here, we speculate that disturbance of intracellular energy metabolism, including glycolysis, by GCs affects sensitivity to cytotoxic anti-cancer reagents.

Previously, we showed that autophagy is a key regulator of cellular energy; it does this by maintaining oxidative phosphorylation (OXPHOS) in the mitochondria, a process essential for ALL cell survival (especially when glycolysis is suppressed) [21]. Autophagy is a self-degradation system in which cytoplasmic components (damaged proteins and organelles) are degraded and recycled by lysosomes. During this process, the isolation membrane (phagophore) sequesters part of the cytoplasm, including abnormal mitochondria and unfolded proteins, to form autophagosomes, which then fuse with lysosomes [22]. In general, cancer cells depend more heavily on autophagy (which is activated by stress) than normal cells to survive [23]. This is because cancer cells experience more acute nutrient and oxygen deprivation due to the higher metabolic demands caused by excessive proliferation [24]. In particular, the oncogenic gene Ras upregulates basal autophagy in several cancers, including pancreatic adenocarcinoma and lung carcinoma, thereby contributing to mitochondrial quality control and maintenance of energy homeostasis when nutrients are lacking [25]. This is in agreement with our previous finding that cancer cells that become under-nourished due to suppression of glycolysis rely on autophagy for energy production.

Here, we examined how the sensitivity of ALL cells to cytotoxic anti-cancer drugs fluctuates when the intracellular energy metabolism is altered by exposure to GCs. In particular, we suggest that GC-mediated suppression of glycolysis activates autophagy to increase mitochondrial function, potentially increasing the cytotoxicity of anti-cancer drugs that bind to the mitochondria. These findings suggest that before we can accurately predict the sensitivity of ALL to anti-cancer drugs, it is necessary to better understand the intracellular pathways that regulate energy metabolism.

## RESULTS

### Combining Dex with anti-cancer drugs enhances anti-cancer effects against some ALL cells

**Figure 1:**
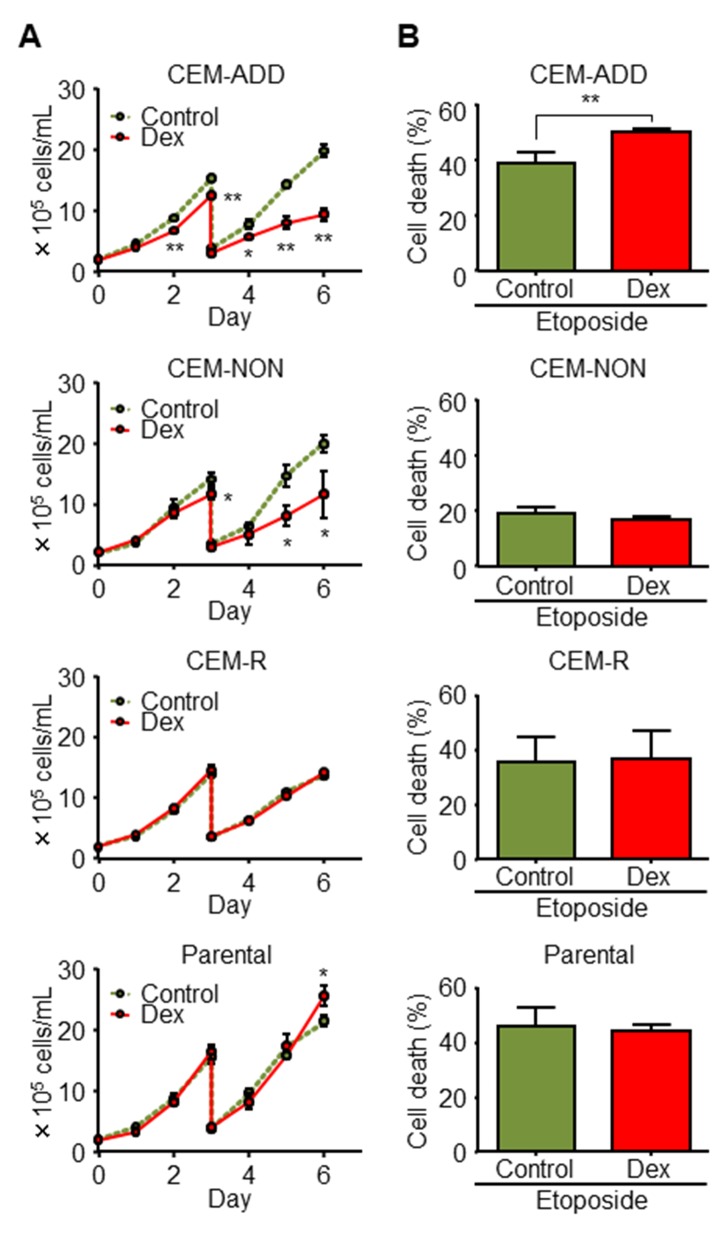
Combined treatment with dexamethasone (Dex) and anti-cancer drugs enhances anti-cancer effects against some acute lymphoblastic leukemia cells **(A)** Isolated CCRF-CEM clones and parental cell lines were cultured in the presence or absence of Dex (1 μM). Cell proliferation was determined by counting the number of cells in each well. Cells were diluted four-fold on Day 3. ^*^P < 0.05 and ^**^P < 0.01, compared with cells cultured in the absence of Dex. **(B)** Each CCRF-CEM clone and the parental cell line was pre-cultured in the presence or absence of Dex for 48 h was treated with etoposide (10 μM) for 72 h. Cell viability was evaluated by flow cytometry. ^**^P < 0.01.

To evaluate the effect of GCs against ALL cells in combination with anti-cancer reagents, we obtained human ALL CCRF-CEM clones and classified them in terms of (i) cytostatic (but not cytotoxic) effects of Dex (a representative GC), and (ii) the combined effects of Dex and a cytotoxic anti-cancer drug (etoposide). We took this approach because CCRF-CEM cells comprise both GC-sensitive or GC-resistant phenotypes [26]. The combined effect of Dex plus etoposide was evaluated by measuring cell death after pre-treatment with Dex. Clones (>20) derived from parental CCRF-CEM cells were classified into three types: 1) shows reduced growth in the presence of Dex and increased etoposide-mediated cytotoxicity in the presence of Dex and etoposide (named CEM-ADD [ADD denotes an “additive” effect of etoposide]); 2) shows notably reduced growth in the presence of Dex, but no increase in cytotoxicity in the presence of etoposide combination (named CEM-NON [“non-additive” effect of etoposide]); and 3) shows no response to Dex, used either alone or in the presence of etoposide (named CEM-R [“resistant” to Dex]) (Figure 1A and 1B). For the parental cells (which comprised various clones), we observed slight Dex-mediated growth suppression, but no increase in cytotoxicity when combined with etoposide (Figure 1A and 1B), suggesting that the number of cells with CEM-ADD-like characteristics determines the overall susceptibility of the parental population to anti-cancer drugs in the presence of GCs.

### Glycolytic inhibition by Dex in CEM-ADD cells increases etoposide-induced cell death

**Figure 2:**
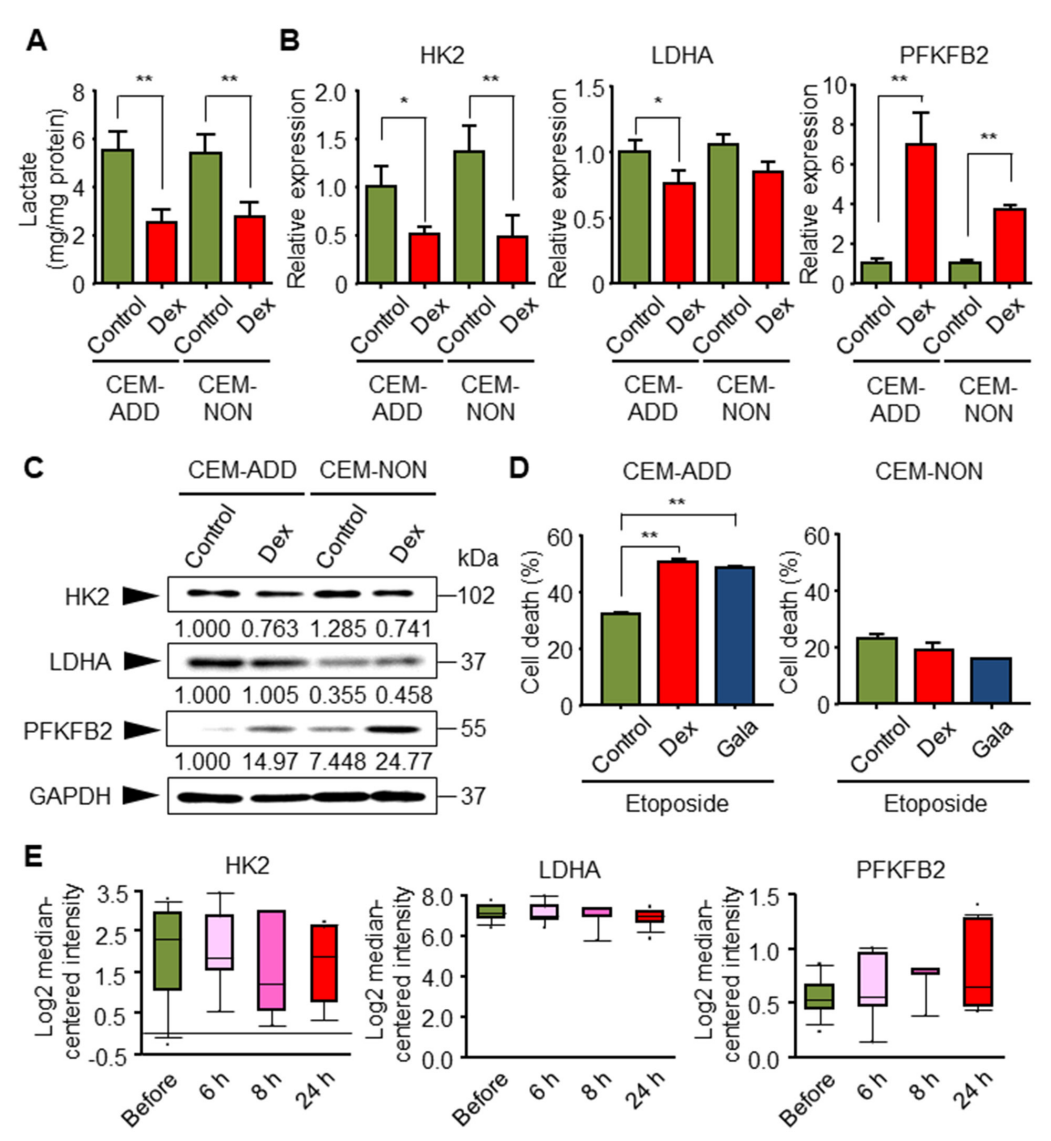
Glycolytic inhibition by dexamethasone (Dex) in CEM-ADD cells leads to increased etoposide-mediated cell death **(A)** CEM-ADD and CEM-NON cells were cultured for 5 days in the presence or absence of Dex (1 μM) and the accumulation of released lactate in the culture medium over the last 3 days was evaluated. ^**^P < 0.01. **(B)** CEM-ADD and CEM-NON cells were cultured in the presence or absence of Dex for 48 h and then subjected to quantitative RT-PCR to measure mRNA encoding hexokinase 2 (HK2), lactate dehydrogenase A (LDHA), and 6-phosphofructo-2-kinase/fructose-2,6-bisphosphatase 2 (PFKFB2). ^*^P < 0.05 and ^**^P < 0.01. **(C)** CEM-ADD and CEM-NON cells were cultured in the presence or absence of Dex for 48 h and then subjected to western blotting with anti-HK2, anti-LDHA, anti-PFKFB2 and anti-GAPDH antibodies. The ratio of the signal intensity of each glycolytic enzyme to that of GAPDH is indicated below each lane. **(D)** CEM-ADD and CEM-NON cells pre-cultured in the presence or absence of Dex, or in galactose (Gala)-based medium, for 48 h were then treated with or without etoposide (10 μM) for 72 h. Cell viability was evaluated by flow cytometry. ^**^P < 0.01. **(E)** Expression of mRNA encoding glycolytic enzymes HK2, LDHA, and PFKFB2, in childhood ALL patients before and at 6 to 24 h after treatment was examined using the Oncomine database (based on the study by Schmidt et al. (2006)).

Next, to explore the reason(s) why GCs increase sensitivity to anti-cancer drugs, we focused on changes in intracellular energy metabolism in the presence of Dex. As mentioned above, GCs suppress the glycolytic pathway [15]; therefore, we speculated that glycolytic suppression by Dex affects sensitivity to etoposide. We confirmed that treatment of CEM-ADD and CEM-NON cells with Dex reduced production of lactate, which is derived from pyruvate during the final step of anaerobic glycolysis (Figure 2A). In addition, and as shown previously [15], mRNA expression of glycolytic enzymes hexokinase 2 (HK2) and lactate dehydrogenase A (LDHA) was reduced, and mRNA expression of an important regulator of glycolytic flux, 6-phosphofructo-2-kinase/fructose-2,6-bisphosphatase 2 (PFKFB2), increased significantly (Figure 2B). Furthermore, protein expression of HK2 and PFKFB2 was also decreased and increased, respectively, but was no clear change in the protein expression of LDHA (Figure 2C).

We previously showed that changing the sugar source in the ALL cell culture medium from glucose to galactose inhibits ATP production via glycolysis [21]. Culturing cells with galactose suppressed growth, but did not induce cell death (data not shown). To investigate whether suppressing glycolysis affects sensitivity to etoposide, we pre-cultured CEM-ADD and CEM-NON cells in galactose-based medium and exposed them to etoposide. We found that the etoposide-sensitivity of CEM-ADD cells pre-cultured in galactose-based medium was similar to that of cells pre-cultured with Dex; however, CEM-NON cells were not affected appreciably (Figure 2D) (see more details in Discussion). These results indicate that GC-mediated enhancement of etoposide-induced cytotoxicity is due to inhibition of glycolysis by GCs.

We next used an extensive expression microarray database derived from cancer patients (Oncomine) [27] to check whether ALL patients treated with GCs show differential expression of glycolysis-related enzymes. Database analysis based on childhood ALL patients indicated that GCs treatment (for 6 to 24 h) tended to reduce the expression of genes encoding HK2 and LDHA, and increase expression of the gene encoding PFKFB2 (Figure 2E); however, these increases/decreases were not significant [28]. These results are consistent with our *in vitro* data.

### Dex induces a metabolic shift from glycolysis to autophagy and mitochondrial OXPHOS

**Figure 3:**
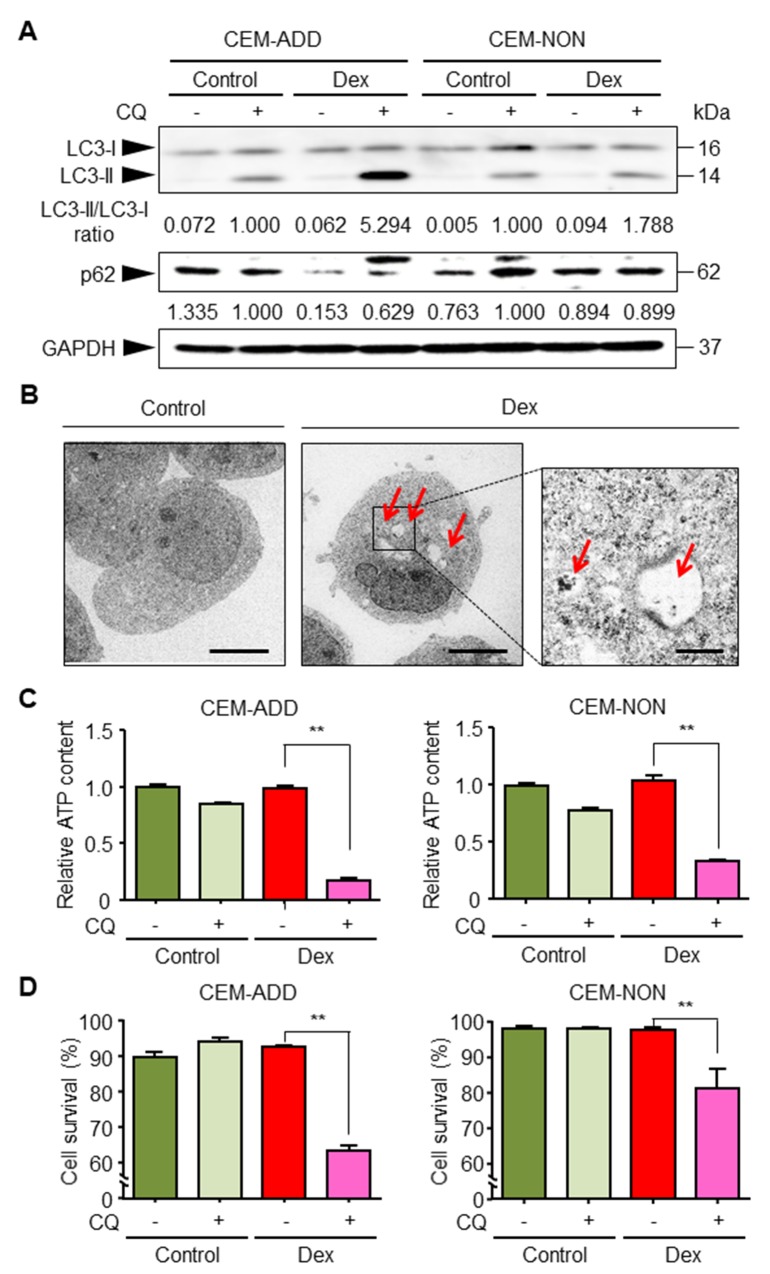
Dexamethasone (Dex) exposure activates autophagy and increases survival of acute lymphoblastic leukemia cells **(A)** Lysates of cells treated with or without Dex (1 μM) or chloroquine (CQ; 20 μM) for 72 h were subjected to western blotting with anti-LC3, anti-p62 and anti-GAPDH antibodies. The ratio of the signal intensity of LC3-II/LC3-I and p62/GAPDH is indicated below each lane. **(B)** After 72 h exposure to Dex, ultrastructural changes in CEM-ADD cells were examined by transmission electron microscopy. Scale bars: 4 μm in the larger panels and 1 μm in the smaller panel. Red arrowheads indicate autophagic bodies. **(C)** CEM-ADD and CEM-NON cells pre-treated with or without Dex for 48 h were then treated with or without CQ (50 μM) for 72 h. The ATP content in the cells was then evaluated. ^**^P < 0.01. **(D)** Viability of the cells in (C) was evaluated using flow cytometry. ^**^P < 0.01.

We previously reported that under conditions that suppress glycolysis, ALL cells activate autophagy to overcome the energy shortfall [21]. Therefore, we assumed that GCs would increase autophagy in ALL cells, thereby increasing sensitivity to anti-cancer drugs. First, we asked whether exposure to Dex increases autophagy in CCRF-CEM cells. Thus, we cultured cells with Dex in the presence/absence chloroquine (CQ; 20 μM), followed by immunoblotting to evaluate changes in a specific marker of autophagy: conversion of LC3-I to LC3-II. CQ inhibits the late steps of autophagic process (fusion of autophagosomes and lysosomes). The results indicated that Dex increased autophagic flux in CEM-ADD cells; however, only a slight increase in autophagy was observed in CEM-NON cells (Figure 3A). Autophagic flux in CEM-ADD cells was also assessed by measuring the decrease in the level of p62 protein, which directly interacts with LC3 and facilitates autophagosome formation, after which it is degraded via autophagy [29]. Figure 3A shows that the decrease in the level of p62 was inhibited by autophagic inhibition with CQ (Figure 3A). In addition, formation of autophagic vesicles and other characteristic structures (i.e., mitophagy), which is indicative of autophagic turnover of mitochondria, were observed under a transmission electron microscope (Figure 3B). Since our previous report argues that ALL cells activate autophagy to overcome the energy shortage caused by inhibited glycolysis [21], we next confirmed whether increased autophagic ability upon Dex treatment increases cell viability. We found that both ATP content (Figure 3C) and viability (Figure 3D) of CEM-ADD and CEM-NON cells pre-cultured with Dex were significantly reduced in the presence of CQ (50 μM); however, the effects of CQ was weaker in CEM-NON cells. These data suggest that exposure to GCs increases autophagy, which in turn supports survival of ALL cells in which glycolysis is suppressed.

**Figure 4:**
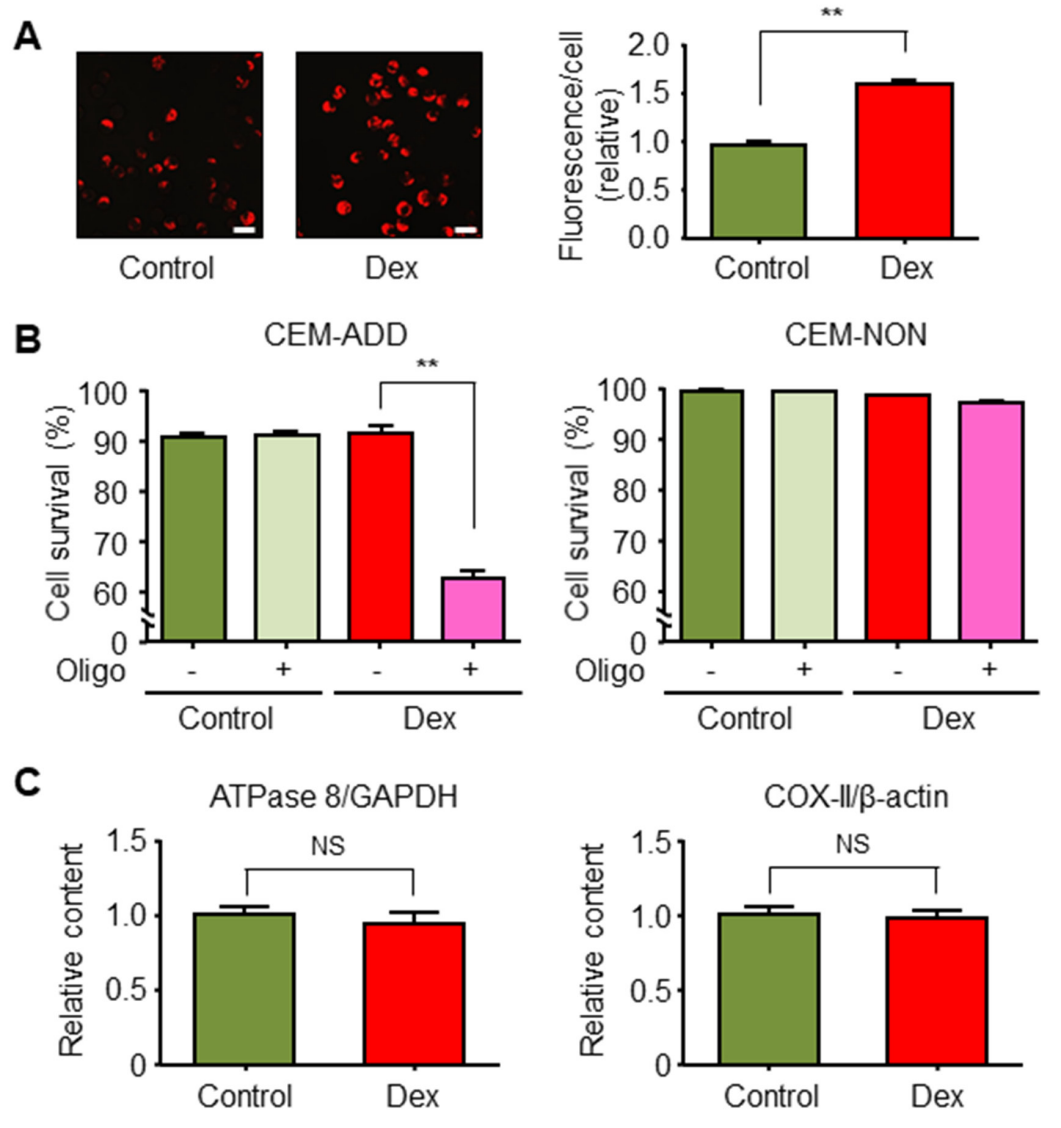
Dexamethasone (Dex) increases mitochondrial function in acute lymphoblastic leukemia cells **(A)** CEM-ADD cells cultured for 72 h in the presence or absence of Dex (1 μM) were stained with MitoTracker Orange and observed under a confocal microscope. Scale bar, 20 μm. Fluorescence intensity per cell was calculated. ^**^P < 0.01. **(B)** CEM-ADD and CEM-NON cells pre-treated with or without Dex for 48 h were then treated with or without oligomycin (Oligo; 25 ng/mL) for 72 h and viability evaluated by flow cytometry. ^**^P < 0.01. **(C)** Mitochondrial DNA, ATPase 8, and cytochrome c oxidase subunit II (COX-II) levels in CEM-ADD cells cultured with or without Dex for 72 h. NS, not significant.

Mitochondrial OXPHOS is an important metabolic pathway for generating large amounts of ATP. We reported previously that ALL cells in which glycolysis is suppressed compensate by deriving energy from mitochondrial OXPHOS [21]. Therefore, we speculated that ALL cells exposed to GCs activate OXPHOS to acquire survival energy. We found that the mitochondrial membrane potential indicated by the high uptake of MitoTracker Orange by mitochondria (Figure 4A). Furthermore, we confirmed that mitochondrial OXPHOS was essential for survival of Dex-treated CEM-ADD cells because the exposure to a specific inhibitor of ATP synthase in mitochondria, oligomycin, led to a significant reduction in cell viability (Figure 4B). In addition, we found that exposure of CEM-ADD cells to Dex did not alter the number of mitochondria (as measured by mitochondrial DNA content, ATPase 8, and cytochrome c oxidase subunit II) (Figure 4C). Taken together, these data suggest that Dex increases mitochondrial turnover to provide the fresh mitochondria required to produce ATP under conditions of reduced glycolysis.

### Dex-induced mitochondrial activation via autophagy in the presence of anti-cancer drugs increases production of reactive oxygen species (ROS)

**Figure 5:**
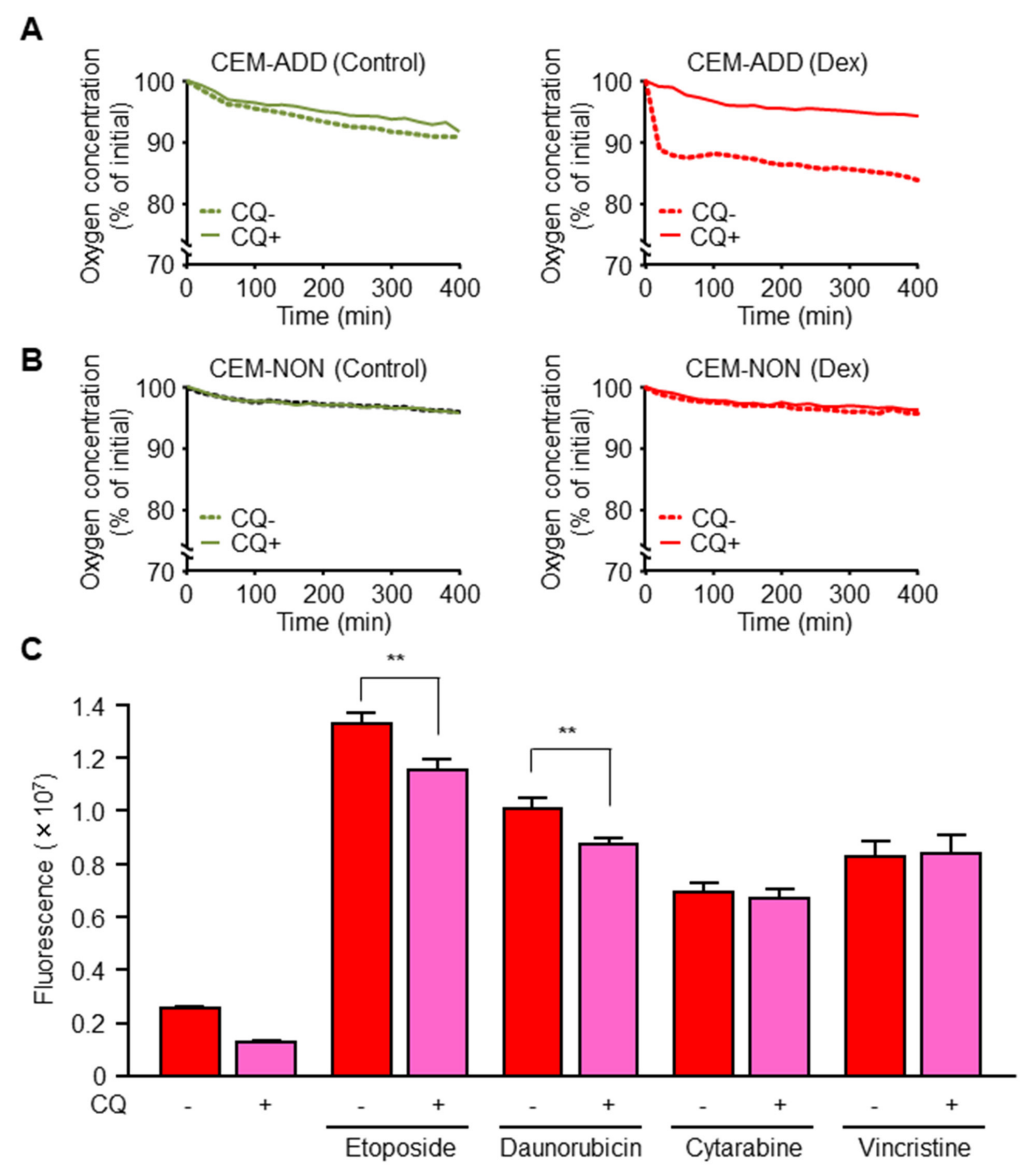
Dexamethasone (Dex)-induced mitochondrial activation via increased autophagy increases anti-cancer drug-mediated reactive oxygen species (ROS) production **(A, B)** Oxygen concentration in the medium of cultured CEM-ADD (A) and CEM-NON cells (B) pre-treated with or without Dex (1 μM) for 72 h was measured over time during additional culture with or without chloroquine (CQ; 20 μM). Data are expressed as the mean of duplicate samples. **(C)** CEM-ADD cells pre-treated with Dex for 48 h were then treated for 24 h with etoposide (10 μM), daunorubicin (100 nM), cytarabine (1 μM), or vincristine (5 nM) in the presence/absence of CQ (20 μM). Intracellular ROS levels were measured by staining with an oxidation-sensitive fluorescent probe dye, 2´,7´-dichlorodihydrofluorescein diacetate. ^**^ P < 0.01.

Mitochondrial OXPHOS generates ROS as a natural byproduct, thereby causing oxidative damage to mitochondrial lipids, cellular DNA, and functional proteins [30]. One system designed to maintain the quality and quantity of mitochondria is mitophagy, which selectively degrades damaged mitochondria [31, 32]. A previous study suggests that autophagy-competent Ras-expressing cancer cells effectively regulate mitochondrial respiration to produce ATP [25]. Therefore, we examined the hypothesis that an increase in mitochondrial function upon Dex exposure is due to increased autophagy. Exposure of CEM-ADD cells (which have the potential to drive autophagy and mitochondrial OXPHOS) to Dex accelerated oxygen consumption from the culture medium; this is an indicator of mitochondrial respiration efficiency (Figure 5A). Inhibition of autophagy by CQ (20 μM) abrogated this effect (Figure 5A). However, exposure of CEM-NON cells to Dex or CQ did not affect the oxygen consumption rate (Figure 5B). These results indicate that Dex-induced autophagy positively regulates mitochondrial function in CEM-ADD cells.

Etoposide and daunorubicin bind to mitochondrial complex-I, one of the respiratory chain complexes [33]. Since complex-I is involved in DNA-damage-induced apoptosis via complex-I-dependent ROS production, we hypothesized that these anti-cancer drugs increase ROS production by mitochondria when mitochondrial biogenesis is enhanced by GCs. In CEM-ADD cells pre-treated with Dex and CQ (20 μM; a low-concentration that did not affect cell viability) reduced ROS production upon exposure to etoposide or daunorubicin (Figure 5C). However, cytarabine and vincristine did not cause a marked change in ROS production in the presence/absence of CQ (Figure 5C). These results indicate that GC-mediated activation of autophagy subsequently increases during mitochondrial biogenesis of ROS through binding of mitochondrial complex-I by anti-cancer drugs.

### Exposure to GCs increases the cytotoxic effects of several anti-cancer drugs against ALL cells

**Figure 6:**
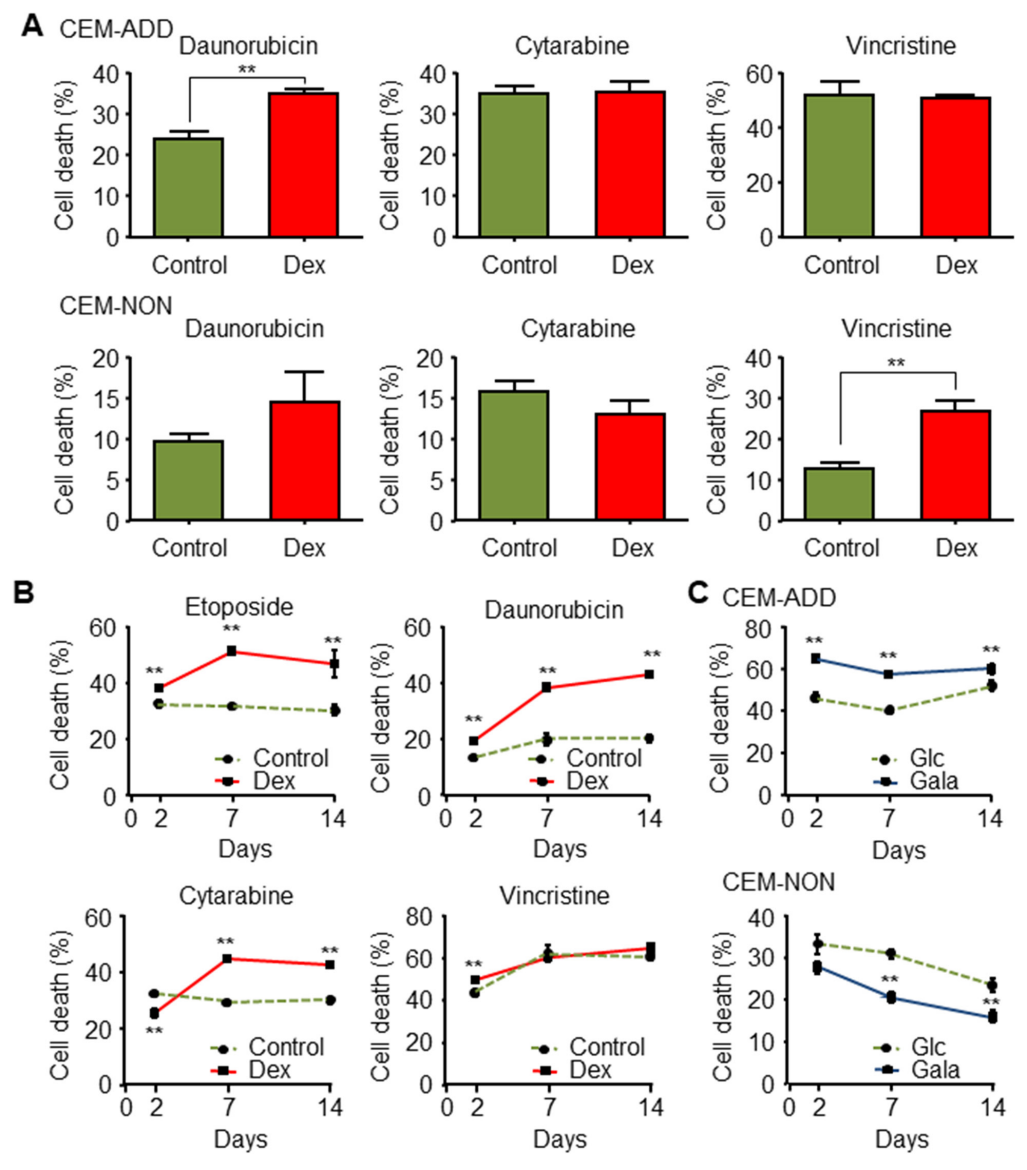
Exposure to glucocorticoids increases the cytotoxic effects of several anti-cancer drugs in acute lymphoblastic leukemia cells **(A)** CEM-ADD and CEM-NON cells pre-cultured in the absence or presence of dexamethasone (Dex; 1 μM) for 48 h were then treated for 72 h with daunorubicin (100 nM), cytarabine (1 μM), or vincristine (5 nM). Cell viability was then measured by flow cytometry. ^**^ P < 0.01. **(B)** CEM-ADD cells pre-cultured in the absence or presence of Dex for 2 days, 1 week, or 2 weeks were then treated with etoposide (10 μM), daunorubicin (100 nM), cytarabine (1μM), or vincristine (5 nM) for 72 h. Cell viability was then evaluated using flow cytometry ^**^ P < 0.01, compared with control cells. **(C)** CEM-ADD and CEM-NON cells pre-cultured in medium containing glucose (Glc) or galactose (Gala) for 2, 7 and 14 days were then treated with etoposide (10 μM) for 72 h. Cell death was then evaluated by flow cytometry ^**^ P < 0.01, compared to cells cultured in Glc-based medium.

In addition to etoposide, we examined the cytotoxic effects of other anti-cancer drugs when combined with Dex. Exposure of CEM-ADD cells to daunorubicin and Dex led to increased ROS production and cell death (Figure 6A). By contrast, cytotoxicity induced by cytarabine or vincristine was almost unchanged in the presence/absence of Dex (Figure 6A). Exposure of CEM-NON cells to vincristine increased cell death in the presence of Dex (the reason for this is unknown), while the cytotoxicity of other anti-cancer drugs did not change significantly in the presence of Dex (Figure 6A). All experiments carried out thus far involved exposing CCRF-CEM cells to Dex for 2 days; therefore, we asked whether pre-treatment with Dex for longer times would increase sensitivity to anti-cancer drugs. As shown in Figure 6B, etoposide and daunorubicin showed greater efficacy when combined with 2, 7, and 14 day pretreatments with Dex, whereas cytarabine-induced cytotoxicity increased when combined with 7 and 14 day treatments with Dex. We then examined whether increased susceptibility to anti-cancer drugs plus Dex was due to a shift in energy metabolism following suppression of glycolysis by Dex. Consistent with the result described in Figure 6B, etoposide was more effective against CCRF-CEM cells that had been cultured for a long time (2, 7 and 14 days) in galactose-based medium than when they were cultured in glucose-based medium (Figure 6C). This suggests that a shift in energy metabolism from glycolysis to mitochondrial OXPHOS via autophagy activity increases the anti-cancer effects of several cytotoxic drugs.

Occasionally, the initiation of cytotoxic therapy in patients with hematologic malignancies, including ALL, increases cancer cell sensitivity [34]. This hypersensitivity induces tumor lysis syndrome (TLS), which is characterized by a group of metabolic derangements caused by massive and abrupt release of cellular components into the blood. In particular, crystallization of uric acid, a metabolite of nucleic acids released from cells, and calcium phosphate may result in impaired renal function, leading to acute renal failure and death in severe cases [34]. Because ALL cells showed increased susceptibility to combination treatment with GCs and anti-cancer drugs, we hypothesized that GCs may play a role in onset of TLS. Analysis of the association between GCs and TLS onset was performed by examining adverse events logged in the Japanese Adverse Drug Event Report database held by the Pharmaceutical and Medical Devices Agency (events were reported from 1 April, 2004, to 31 August, 2014; 309,015 cases). We examined effects related to Dex, prednisolone, hydrocortisone and fluticasone. The reported number of adverse drug reactions for ALL patients was 711 and 398 for GCs and non-GCs, respectively. Of these, the number of TLS cases was 15 and 1, respectively. We calculated the reporting odds ratio (ROR), a measure of the relative risk for drug-associated adverse events [35], and found a 95% confidential interval (CI) (two-sided) of 8.556 and 1.125–65.02, respectively. The lower limit of the ROR (95% CI) was >1, suggesting that administration of GCs may be associated with TLS onset caused by excessive sensitivity to anti-cancer drugs.

## DISCUSSION

Our previous study showed that autophagy drives mitochondrial OXPHOS under glycolysis-suppressed conditions [21]. Here, we showed that GC-mediated suppression of glycolysis in ALL cells switches energy metabolism from glycolysis to autophagy and mitochondrial OXPHOS. Cancer cells mainly obtain energy (ATP) for survival via glycolysis rather than via mitochondrial OXPHOS, even in the presence of available oxygen (a process known as the “Warburg effect”) [36]. In addition, recent studies reveal that reprogrammed mitochondrial uncoupling in leukemia cells promotes the Warburg effect [37]. However, suppressing glycolysis alone does not eradicate tumors *in vivo* [20]; this is supported by our previous *in vitro* study of leukemia cells [21]. Therefore, cancer cells must have an alternative pathway for obtaining survival energy under conditions of suppressed glycolysis. Thus, we focused on mitochondrial OXPHOS as the alternative pathway and speculated that activity is maintained via mitophagy. Mitophagy, selective degradation of mitochondria via the autophagic pathway, contributes to mitochondrial quality control by removing dysfunctional mitochondria [38]. Also, mitochondrial turnover via mitophagy plays an important role in maintaining the activity of respiratory supercomplexes [39]. Here, we showed that exposing CEM-ADD cells to Dex increased the mitochondrial membrane potential without increasing the number of intracellular mitochondria. Previously, Samuels et al. have reported that ALL cells directly damaged by GCs are highly dependent on glycolysis for survival and proliferation [40]. In ALL cells, exposure to GCs suppresses glycolysis (but does not induce cell death) and increases mitochondrial bioenergetics. This is supported by the observation that combined treatment with oligomycin and GCs is effective in reducing viability [40]. From their analyses, we can speculate that ALL cells, which are highly dependent on glycolysis, have the potential to shift between glucose bioenergetic pathways (i.e., between glycolysis and mitochondrial OXPHOS). We also found that the metabolic shift is mediated by autophagy. Taken together, these results suggest that Dex-induced mitophagy alters the metabolic system in ALL cells, thereby improving the quality of mitochondria that can then support energy acquisition in ALL cells exposed to glycolytic suppression.

During ALL therapy, GCs are often used in combination with cytotoxic anti-cancer drugs [4]. Here, we showed that a combination of Dex plus anti-cancer drugs that bind mitochondria increases the efficacy of anti-cancer drugs; this increase is due to increased production of ROS. The results show that exposure to etoposide and daunorubicin increased ROS production by inhibiting mitochondrial complex-I, particularly when mitochondrial biogenesis is enhanced by mitophagy. This scenario is supported by the following reports showing that hyperpolarizing mitochondrial membrane potential stimulates ROS production [41], and that mitochondrial impairment of OXPHOS complexes generates large amounts of ROS [42]. However, autophagic inhibition (probably mitophagic inhibition) by CQ in the presence of etoposide or daunorubicin led to a small but significant reduction of ROS levels in Dex-treated CCRF-CEM cells. We must further consider the following points: 1) other pathways, such as those induced by anti-cancer drugs themselves [43], and poly ADP-ribose polymerase and NAD(P)H oxidase also generate intracellular ROS after anti-cancer drug-induced DNA-damage [44]); and 2) autophagy, driven by various intracellular stresses (including ROS), plays a role in eliminating these stresses [45]. Thus, inhibition of autophagy may also increase intracellular ROS levels by preventing elimination of ROS generated by anti-cancer drugs. Therefore, to predict sensitivity of ALL to anti-cancer drugs plus GCs, we need to consider both the increase in ROS production due to inhibition of mitochondrial function by anti-cancer drugs or by the anti-cancer drugs themselves, and removal of intracellular stresses by autophagy.

To examine susceptibility ALL cells to GCs, many researchers used cloned cells [26, 46]. We also cloned CCRF-CEM cells to examine individual differences in reactivity to GCs and anti-cancer drugs. Among the cell populations that entered cytostasis upon exposure to Dex, we observed that some did and some did not show increased sensitivity to anti-cancer drugs in the presence of (CEM-ADD and CEM-NON cells, respectively). Therefore, we examined differences in energy metabolism under conditions of Dex-mediated glycolytic suppression. CEM-ADD cells were highly dependent on alternative energy acquisition pathways (autophagy and mitochondrial OXPHOS); however, CEM-NON cells had low energy demands under the same conditions. A study based on tumor-derived cell lines from the National Cancer Institute showed that metabolic activity of cancer cells is coupled to their cell size and rate of protein synthesis [47]. The cells survive by keeping the requirement for ATP low under stress conditions, such as starvation. We also speculate that CEM-NON cells require less ATP under glycolysis-suppressed conditions induced by Dex exposure; thus, Dex did not affect mitochondrial function in CEM-NON cells or their sensitivity to anti-cancer drugs. In addition, many human tumors such as breast and prostate cancers show defective autophagy; one of the main reasons for this is the lack of essential autophagic genes [48, 49]. Besides loss of function of autophagy in cancer cells, increased survival and proliferative signals, such as PI-3 kinase and mTOR, render them less able to induce autophagy [50]. We could not identify the precise mechanism underlying the finding that CEM-NON cells do not rely on autophagy to a great extent, but we believe potential differences in autophagic and mitochondrial function alter cellular sensitivity to anti-cancer drugs in the presence of GC.

**Figure 7:**
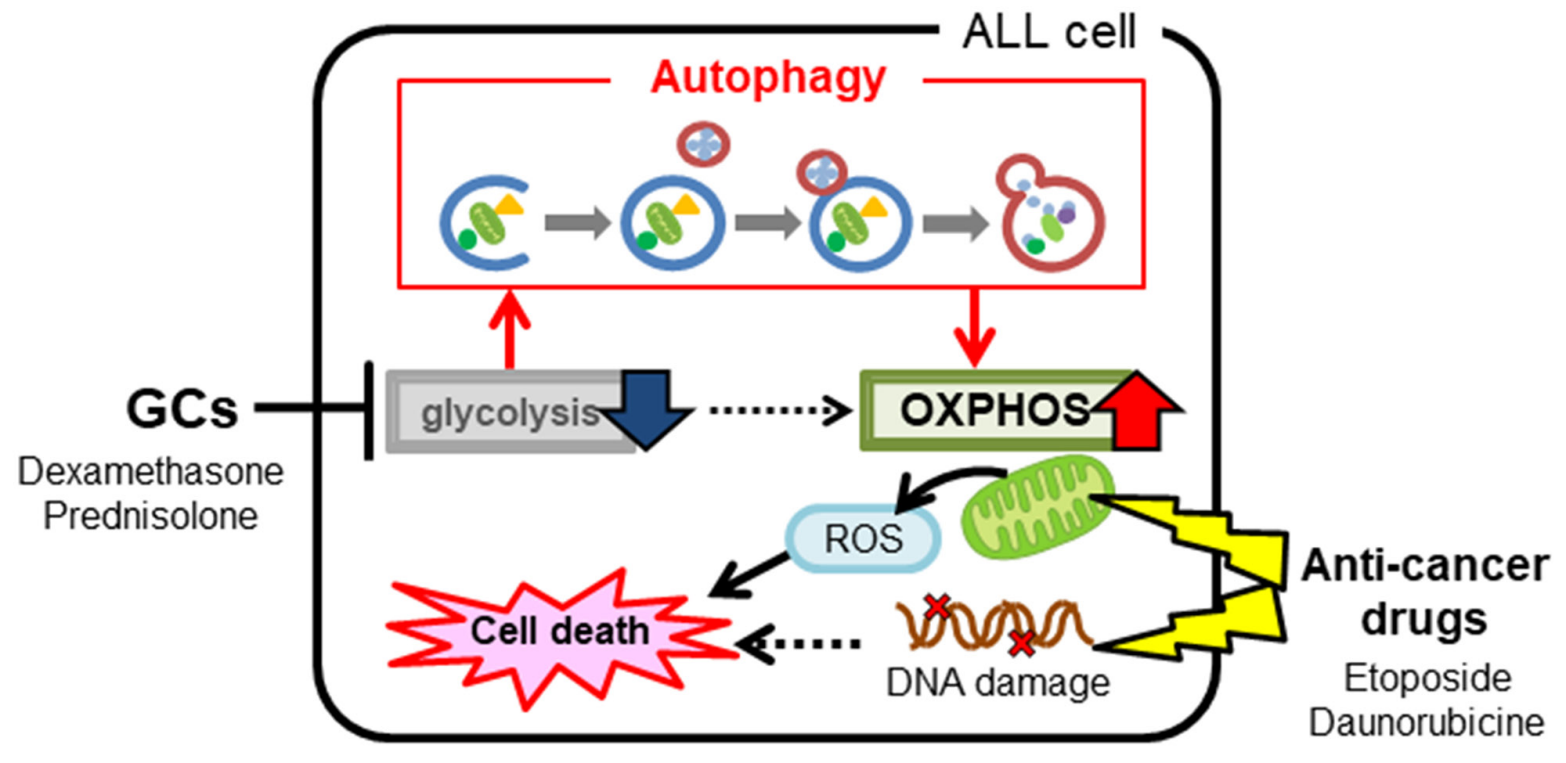
An overview of the study findings Administration of glucocorticoids to acute lymphoblastic leukemia cells alters intracellular energy metabolism by suppressing glycolysis and activating mitochondrial function via autophagy. Altered metabolism affects the efficacy of concomitant anti-cancer drugs, particularly those capable of inhibiting mitochondrial function.

In the clinic, a standard protocol is to test the response of ALL cells to GCs and then determine the treatment regimen [7]. However, this method does not provide sufficient information about how GC administration affects ALL sensitivity to anti-cancer drugs. Therefore, it is difficult to predict accurately the effect of combination therapy. Here, we suggest that there is a cause-and-effect relationship between onset of TLS, a serious side effect associated with excessive susceptibility to anti-cancer drugs, and the use of GCs. Since severe TLS can lead to acute renal failure and death, it is very important that we develop a method that can predict sensitivity to anti-cancer drugs when used in combination with GCs. The results presented herein suggest that differences in susceptibility of ALL cells to combined treatment with anti-cancer drugs and GCs is due to differences in mitochondrial activity and autophagic function. We suggest that individual differences in mitochondrial function, and in the ability of cells to induce autophagy, in the presence of GCs determines susceptibility to anti-cancer drugs. Thus, anti-cancer drugs should be selected after considering alterations in intracellular energy metabolism caused by GCs; this will improve the safety and efficacy of ALL treatment. An overview of the findings of our study is shown in Figure 7.

## MATELIALS AND METHODS

### Reagents

CQ (Sigma, St. Louis, MO, USA) was dissolved in Milli Q water. Oligomycin (Sigma), Dex (Wako, Tokyo, Japan), etoposide (Tokyo Kasei, Tokyo, Japan), daunorubicin (Cayman Chemical, MI, USA), cytarabine (LKT Laboratories, St. Paul, MN, USA) and vincristine (LKT Laboratories) were dissolved in dimethyl sulfoxide (DMSO). The final DMSO concentration in every cell culture did not exceed 0.5% (v/v).

### Cell culture and isolation of clones

CCRF-CEM cells, purchased from RIKEN Cell Bank (Tsukuba, Japan), were maintained in RPMI-1640 (Nacalai Tesque, Kyoto, Japan) supplemented with 10% fetal bovine serum (FBS, Life Technologies, Grand Island, NY, USA) plus antibiotics (Nacalai Tesque) at 37°C in a humidified atmosphere of 5% CO_2_ in air. For each experiment, cells were cultured in medium containing glucose (2 g/L) or galactose (2 g/L). To isolate clones, 100 μL of cell suspension was added to each well of a 96-well plate (1 cell per well). After several days, growing cells derived from a single cell were transferred to 12-well plate and/or a larger dish-plate for expansion culture.

### Detection of dead cells by flow cytometry

To distinguish dead cells from live cells, CCRF-CEM cells were stained with the Zombie NIR™ Fixable Viability kit (BioLegend, San Diego, CA, USA), according to the manufacturer’s instructions, and examined in a cell analyzer (EC800; SONY, Tokyo, Japan).

### Measurement of cellular lactate release

Release of cellular lactate was measured as described previously [21]. Briefly, the supernatant from cultured cells was de-proteinized with perchloric acid and neutralized with potassium hydroxide. The supernatant was then mixed with nicotinamide adenine dinucleotide and glutamate pyruvate transaminase (Roche, Mannheim, Germany). The enzymatic reaction was started by adding lactate dehydrogenase (Wako) to each sample at 37°C for 30 min. Absorbance was the measured at a wavelength of 340 nm.

### RNA isolation and quantitative real-time PCR

Total RNA was isolated from CCRF-CEM cells using Sepasol-RNA I reagent (Nacalai Tesque) and reverse transcribed using ReverTra Ace^®^ qPCR RT Master mix (TOYOBO, Osaka, Japan). The resulting cDNA was mixed with THUNDERBIRD™ quantitative real-time PCR mix (TOYOBO) and subjected to quantitative real-time PCR using a LightCycler™ Nano Real-Time PCR System (Roche) and the following primers: HK2 forward, 5´-ACA GGT GCT CTC AAG CCC TAA G-3´ and reverse, 5´-CGA GGC CGC CAT CTC AGA GCG G-3´; LDHA forward, 5´-GGA GAT CCA TCA TCT CTC C-3´ and reverse, 5´-GGC CTG TGC CAT CAG TAT CT-3´; PFKFB2 forward, 5´-GAT TGG AGT ACC CAC CAA AGT G-3´ and reverse, 5´-TTC ACG TCG ATA TAC CCC AAG A-3´; and β-actin forward, 5´-TTC AAC ACC CCA GCC ATG TAC G-3´ and reverse, 5´-GTG GTG GTG AAG CTG TAG CC-3´. The cycling conditions were as follows: 95°C for 60 s, followed by 40 cycles at 95°C for 10 s and 60°C for 60 s. Relative expression of mRNA was calculated after normalization against β-actin.

### Autophagic flux assay and western blot analysis

Autophagic flux was evaluated by monitoring turnover of the autophagic marker LC3-II by western blot analysis in the presence and absence of CQ [12]. Autophagic flux was measured as the difference in LC3-II protein expression between CQ-treated samples and CQ-untreated samples in the respective group. For western blot analysis, cells were harvested and lysed on ice in cell lysis buffer containing phosphate-buffered saline (pH 7.4), 1% Triton X-100, and a protease inhibitor cocktail (Roche). Next, 5 μg of protein from each sample was loaded and run on SDS-PAGE gels and transferred to polyvinylidene difluoride membranes (Merck Millipore, Berlin, Germany). After blocking with 5% bovine serum albumin, the membranes were probed with specific primary antibodies (Cell Signaling Technologies, Beverly, MA, USA), according to the manufacturer’s instructions. Immunolabeled proteins were detected using a horseradish peroxidase-labeled donkey anti-rabbit IgG secondary antibody (Santa Cruz Biotechnology, Santa Cruz, CA, USA) and the ECL prime detection reagent (GE Healthcare, Buckinghamshire, UK).

### Transmission electron microscopy

Cells were prepared for transmission electron microscopy and observed as previously described [51–53]. Briefly, CEM-ADD cells were fixed by addition of 2.5% (final) glutaraldehyde to the culture medium for 24 h on ice. Glutaraldehyde-fixed samples were then sandwiched between two copper discs, snap-frozen with melting propane, cooled in liquid nitrogen, and freeze-substituted in acetone containing 2% osmium tetroxide at -80°C for 2 days. Finally, samples were embedded in an epoxy resin. Ultrathin sections (70 nm thick) were cut, stained with uranyl acetate and lead citrate, and observed under a JEM-1400 electron microscope (JEOL, Tokyo, Japan) at nominal magnifications of 2,500–10,000.

### Measurement of the cellular ATP content

The CellTiter-Glo Luminescent Cell Viability Assay (Promega, Madison, WI, USA) was used to measure cellular ATP content in suspensions of CCRF-CEM cells, according to the manufacturer’s instructions.

### Evaluation of mitochondrial membrane potential

To evaluate mitochondrial membrane potential, CCRF-CEM cells were incubated for 45 min with 200 nM MitoTracker Orange (Life Technologies) dissolved in FBS-free RPMI-1640. After loading, cells were washed in PBS and images obtained using a Carl Zeiss LSM700 laser scanning confocal microscope (Prenzlauer, Berlin, Germany). Fluorescence intensity and cell number were quantified using Image J (NIH, Bethesda, MD).

### Analysis of mitochondrial DNA content

Mitochondrial DNA content was quantified as described previously [54]. Briefly, total cellular DNA (both mitochondrial and nuclear DNA) was isolated using the NucleoSpin^®^ Tissue kit (Macherey Nagel, Düren, Germany). Total DNA was subjected to quantitative real-time PCR using the following primers: mitochondrial ATPase 8 forward, 5´-AAT ATT AAA CAC AAA CTA CCA CCT ACC-3´ and reverse, 5´-TGG TTC TCA GGG TTT GTT ATA-3´; mitochondrial cytochrome c oxidase subunit II forward, 5´-CCC CAC ATT AGG CTT AAA AAC AGA T-3´ and reverse, 5´-TAT ACC CCC GGT CGT GTA GCG GT-3´; nuclear GAPDH forward, 5´-AAG GTC ATC CCT GAG CTG AA-3´ and reverse, 5´-TTC TAG ACG GCA GGT CAG GT-3´; and nuclear β-actin forward and reverse. Relative amounts of mitochondrial DNA in cells were calculated after normalizing against either nuclear GAPDH or β-actin DNA.

### Measurement of cellular oxygen consumption rate

The oxygen consumption of CCRF-CEM cells was measured using a fluorescent oxygen probe (PreSens Sensor Dish Reader; Regensburg, Germany). Oxygen tension was monitored continuously every minute and the concentration at time 0 was set to 100%.

### Measurement of intracellular ROS

Intracellular ROS was measured in CEM-ADD cells using an oxidation-sensitive fluorescent probe dye: 2´,7´-dichlorodihydrofluorescein diacetate (DCF-DA, Life Technologies) [55]. Briefly, CEM-ADD cells treated with anti-cancer drugs were incubated for 45 min with 25 μM DCF-DA dissolved in HBSS. After loading, cells were washed and fluorescence generated by 2′,7′-dichlorofluorescein measured at excitation/emission wave lengths of 485/535 nm (Molecular Devices, Sunnyvale, CA, USA).

### Statistical analysis

All data were expressed as the mean ± SD of at least three independent experiments unless indicated otherwise. Statistical analysis was performed using Student’s *t* test or analysis of variance followed by the Bonferroni test where applicable. A p value of < 0.05 was considered significant.

## Abbrevations

ALL: acute lymphoblastic leukemia
CQ: chloroquine
DCF-DA: 2´,7´-dichlorodihydrofluorescein diacetate
Dex: dexamethasone
GCs: glucocorticoids
HK2: hexokinase 2
LDHA: lactate dehydrogenase A
OXPHOS: oxidative phosphorylation
PFKFB2: 6-phosphofructo-2-kinase/fructose-2,6-bisphosphatase 2
ROR: reporting odds ratio
ROS: reactive oxygen species
TLS: tumor lysis syndrome
